# A Genetically Engineered *Escherichia coli* for Potential Utilization in Fungal Smut Disease Control

**DOI:** 10.3390/microorganisms11061564

**Published:** 2023-06-13

**Authors:** Guobing Cui, Xinping Bi, Shan Lu, Zide Jiang, Yizhen Deng

**Affiliations:** 1 Guangdong Laboratory for Lingnan Modern Agriculture, South China Agricultural University, Guangzhou 510642, China; 2 Henry Fork School of Biology and Agriculture, Shaoguan University, Shaoguan 512000, China; 3 State Key Laboratory for Conservation and Utilization of Subtropical Agro-Bioresources, Guangdong Province Key Laboratory of Microbial Signals and Disease Control, Integrative Microbiology Research Centre, South China Agricultural University, Guangzhou 510642, China; 4 State Key Laboratory for Conservation and Utilization of Subtropical Ago-Bioresouces Ministry and Province Co-Sponsored Collaborative Innovation Center for Sugarcane and Sugar Industry, Nanning 530004, China

**Keywords:** filamentous growth, jasmonic acid carboxyl methyl transferase (JMT), methyl jasmonate (MeJA), pathogenicity, *Sporisorium scitamineum*, sugarcane smut

## Abstract

*Sporisorium scitamineum*, the basidiomycetous fungus that causes sugarcane smut and leads to severe losses in sugarcane quantity and quality, undergoes sexual mating to form dikaryotic hyphae capable of invading the host cane. Therefore, suppressing dikaryotic hyphae formation would potentially be an effective way to prevent host infection by the smut fungus, and the following disease symptom developments. The phytohormone methyl jasmonate (MeJA) has been shown to induce plant defenses against insects and microbial pathogens. In this study, we will verify that the exogenous addition of MeJA-suppressed dikaryotic hyphae formation in *S. scitamineum* and *Ustilago maydis* under in vitro culture conditions, and the maize smut symptom caused by *U. maydis,* could be effectively suppressed by MeJA in a pot experiment. We constructed an *Escherichia coli*-expressing plant *JMT* gene, encoding a jasmonic acid carboxyl methyl transferase that catalyzes conversion from jasmonic acid (JA) to MeJA. By GC-MS, we will confirm that the transformed *E. coli*, designated as the pJMT strain, was able to produce MeJA in the presence of JA and S-adenosyl-L-methionine (SAM as methyl donor). Furthermore, the pJMT strain was able to suppress *S. scitamineum* filamentous growth under in vitro culture conditions. It waits to further optimize *JMT* expression under field conditions in order to utilize the pJMT strain as a biocontrol agent (BCA) of sugarcane smut disease. Overall, our study provides a potentially novel method for controlling crop fungal diseases by boosting phytohormone biosynthesis.

## 1. Introduction

*Sporisorium scitamineum* is the fungal pathogen that causes sugarcane smut [1]. Diploid teliospores of *S. scitamineum* are airborne and can survive in harsh environmental conditions. The smut teliospores germinate on sugarcane buds, forming promycelium from which two pairs of haploid sporidia (basidiospores) with opposite mating types, *MAT-1* and *MAT-2*, are generated via a round of meiosis. The sporidia of opposite mating types can recognize each other and fuse, undergoing sexual mating, and afterwards switch from a yeast-like growth style to dikaryotic hyphae, undergoing a so-called dimorphic switch. Dikaryotic hyphae are capable of infecting sugarcane, likely through buds. The smut disease symptoms are characterized by the emergence of a typical structure produced on the apex or side shoots of sugarcane stalks, called “smut whip”, which contains teliospores formed from a fusion of two nuclei of the dikaryotic hyphae [2,3].

Given that a post-mating dimorphic switch is critical for host infection, molecules controlling this step are potential disease management targets. The mechanisms of sexual mating and pathogenicity of smut fungi were well-studied on the model fungus *Ustilago maydis* [4]. The processes of infection and sexual mating are similar in smut fungus, and the research on *U. maydis* provides an important reference for *S. scitamineum* [5]. Different mating types are determined by two alleles, named *MAT* loci which include a locus and b locus, in *S. scitamineum* and *U. maydis* [5]. In *S. scitamineum,* the *MFA* genes of the mating locus encode pheromone precursors (including a factor and α factor), which undergo a series of post-translational modifications and are secreted out of sporidial cells, serving as signaling molecules [6,7,8]. The a or α pheromone is recognized by the pheromone receptor Pra of the opposite-mating type, which transmits the signal into the cell and through the MAPK or cAMP-PKA, and thus signaling cascades to the global transcriptional factor Prf1, which in turn induces dikaryotic hyphae growth via activating a and b locus [8,9,10,11]. The conserved farnesyltransferase (FTase) β subunit SsRam regulates the yeast-to-hyphae dimorphic switch, stress resistance, and pathogenicity of *S. scitamineum*, likely via catalyzing farnesylation of the pheromone precursor Mfa1 [8]. Besides conveying the sexual pheromone signal, the MAPK SsKpp2 and SsHog1 are involved in the response to extracellular osmotic stress, and thus regulate the dimorphic switch and stress resistance [9,11]. An investigation of the molecular mechanism underlying the *S. scitamineum* dimorphic switch/filamentous growth would provide a theoretical basis for developing novel and effective management strategies of sugarcane smut, which is largely lacking and urgently needed in agricultural practices.

Jasmonic acid (JA) and its methylated product, methyl jasmonate (MeJA), are important phytohormones and play an important role in plant resistance to biotic and abiotic stresses [12]. MeJA treatment with the tomato before sowing can reduce the attack of the soil-born fungal pathogen *Fusarium oxysporum* f.sp. *lycopersici* [13]. Endogenous MeJA content of plants increases significantly in response to mechanical damage, pathogen infection, ozone and metal stress in *Brassica napus* L. [14]. In *Arabidopsis thaliana*, gaseous MeJA can effectively reduce the harm caused by fungal pathogens *Alternaria brassicicola*, *Botrytis cinerea,* or *Plectosphaerella cucumerina* [15]. Jasmonic acid carboxyl methyl transferase (JMT) catalyzes MeJA formation by using JA as a precursor [16]. Over-expression of *AtJMT* in *A. thaliana* induces the constitutive expression of JA biosynthesis-related genes and confers enhanced resistance towards the fungal pathogen *B. cinerea* [17]. Rice *OsJMT* gene is induced by infestation with brown planthopper (*Nilaparvata lugens*), catalyzing JA to MeJA and thus regulating herbivore-induced defense responses [18]. Taken together, enhanced levels of MeJA by JMT function can confer plant immunity against pathogenic fungi and pests.

In this study, MeJA displayed a significant inhibitory effect on *S. scitamineum* mating/filamentation, while JA did not. In addition, exogenous application of MeJA to maize seedlings grown in pots could significantly improve the ability of maize to resist the occurrence of smut disease. Through heterologous expression of *A. thaliana JMT* gene in *Escherichia coli*, we generated a strain, designated as pJMT strain, capable of converting JA to MeJA under in vitro culture conditions. To test the capacity of pJMT strain in controlling smut disease, we applied it to maize or sugarcane plants exposed to smut fungi (*U. maydis* and *S. scitamineum*), respectively. In summary, enhancing plant resistance to pathogens by microbes-dependent phytohormone biosynthesis may serve as a biological control method pending necessary optimization for utilization in field conditions.

## 2. Materials and Methods

### 2.1. Fungal Strains and Culture Conditions

The *S. scitamineum* strains *MAT-1* (eGFP) and *MAT-2* (dsRED) used in this study were generated by Yan et al. [19], and stored in Deng Y’s lab. The haploid *MAT-1* (U9) and *MAT-2* (U10) strains of *U. maydis* were provided by Jiang Z’s lab. The *S. scitamineum* and *U. maydis* sporidia were cultured in YePS medium [19], and the sexual mating medium of *U. maydis* was YePS medium supplemented with 1% activated carbon. Fungal cells were cultured in 2 mL YePS medium at 28 °C, shaken at 200 rpm for 2 days, then collected by centrifugation and washed with 2 mL sterilized water before being re-suspended in sterilized water to reach OD_600_ ≈ 1.0. Equal volumes of *S. scitamineum* or *U. maydis* sporidia, of opposite mating types, were mixed to induce sexual mating and the following filamentous growth. For the suppression assay, sporidia of wild-type *MAT-1* and *MAT-2* sporidia of *S. scitamineum* (OD_600_ ≈ 1.0 for both) were mixed (1 μL for each) and allowed to grow on YePS solid medium containing JA or MeJA of different concentrations. The filamentation of the fungal colonies was assessed visually, and the experiments were repeated three times.

### 2.2. Construction of JMT Expression Plasmid

The *A. thaliana JMT* (GeneBank: AY008435.1) nucleotide sequence was synthesized by Suzhou Genewiz Biological Technology Co., Ltd. (Suzhou, China), and inserted into the *EcoR* I-*Sal* I site of the expression vector pRSFDUET-1 [20]. The resultant plasmid carries the *JMT* gene driven by T7 promoter following the lacI repressor, and therefore is inducible by Isopropyl β-D-Thiogalactoside (IPTG) [21]. The expression plasmid was transformed into *E. coli* BL21(DE3) strain, and verified by PCR amplification using the primers as listed in Table 1. The verified strain was named pJMT strain. To test whether it could produce MeJA, JA (as the precursor) and SAM (S-adenosyl-L-methionine as methyl donor) were applied to the culture medium.

### 2.3. Total RNA Extraction and Quantitative Real-Time PCR (qRT-PCR)

A RNeasy mini kit (Qiagen (Hilden, Germany), 74104) was used for total RNA extraction. A TURBO DNA-free kit (Invitrogen (Waltham, MA, USA), AM1907) and TransScript first-strand cDNA synthesis supermix (TransGen Biotech (Beijing, China), AT301) were, respectively, used for gDNA removal and first strand cDNA synthesis, following manufacturer’s instructions. PowerUp SYBR green master mix (Applied Biosystems (Waltham, MA, USA), A25742) was used for qRT-PCR, with the primers as listed in Table 1, and the reaction was run on a Quant Studio 6 Flex Real-Time PCR system (Applied Biosystems). Relative expression folds were calculated by the 2^−ΔΔCT^ method [22] with *ACTIN* as an internal control. Statistical analysis was performed by one-way ANOVA and Duncan’s multiple analyses.

### 2.4. JMT Gene Induction

Induced expression of *JMT* gene: The pJMT-carrying strain (hereafter named as pJMT strain) was cultured in 5 mL LB broth containing 50 μg/mL kanamycin (Dingguo, AK177), at 37 °C and shaken at 200 rpm, until OD_600_ ≈ 1.0. IPTG (Genview, CI175) was added to the pJMT strain culture to reach the final concentration of 1 mM, and then it was cultured at 28 °C and 200 rpm for another 12 h. The bacterial cells were collected by centrifugation at 1000× *g* and transferred to 50 mL of LB medium containing 200 μM jasmonic acid (JA, Sigma-aldrich (St. Louis, MO, USA), J2500) and 200 μM SAM (Sigma-aldrich, A4377), and incubated at 28 °C, 200 rpm for 12 h. The *E. coli* expressing the vector pRSFDUET-1 was used as a negative control.

### 2.5. MeJA or JA Detection

For the extraction of MeJA from the induced pJMT strain cultured in the presence of JA and SAM, the supernatant was collected after centrifuge and mixed with ethyl acetate of equal volume. After phase separation, the ethyl acetate phase was collected and dried by rotary evaporation (EYELA, OSB-2100). Such extracts were dissolved in 1 mL of methanol and filtered through a 0.22-μm microporous filter (Nylon6). For detection of JA from the extracts, high performance liquid chromatography (HPLC) was performed using the following settings: 0.1% formic acid; water = 65/35 (*v*/*v*); flow rate = 0.3 mL/min; detection wavelength = 205 nm; detection time = 20 min; and column temperature = 30 °C. The column is Agilent HC-C18(2) 250 × 4.6 mm (5 μm).

MeJA was identified by gas chromatography/mass spectrometry (GC/MS) on an Agilent 7890B/5977B (Agilent Technologies Inc., Santa Clara, CA, USA) series GC system equipped with an Agilent HP-5MS capillary column (30 m × 250 µm × 0.25 µm) in scan mode and split mode. The following temperature program was used: initial temperature of 60 °C (5 min hold), increase to 270 °C at 30 °C/min. Ion source temperature was 220 °C, and transfer line temperature was 270 °C. Electron ionization energy was 70 eV, and m/z = 30~450. MeJA was identified through standard compounds from the National Institute of Standards and Technology (NIST, Gaithersburg, MD, USA) library database.

### 2.6. Pathogenicity Assays

Pot experiment: Maize (Hua Meitian 9; DYQS-16 × TG-9) was grown in pots until the four-leaf stage, under the growth condition set as follows: humidity: 82%; light duration: 12 h. The mixed *U. maydis MAT-1* and *MAT-2* cells (OD_600_ ≈ 1.0, 1:1 in volume), with or without 400 μM MeJA, were injected to the base of maize leaves. Injection with sterile water served as blank control. The injected maize seedlings were allowed to grow under natural conditions for 3 weeks, before examination of disease symptoms and photographing. The pJMT strain’s effect on maize smut disease was tested following the same procedure. The pJMT strain was pre-treated with IPTG to induce expression of the *JMT* gene.

Field experiment: Approximately 290 seedlings of ratoon cane (ROC 22) were used for pJMT treatment, or untreated as control, respectively. The sugarcane cultivar ROC 22 was established as a susceptible cultivar towards smut fungus, as reported [23]. The pathogenicity and biocontrol assay were carried out on an established diseased field (Experimental fields of South China Agricultural University, 23.23 E, 113.63 W), which steadily causes smut disease to the susceptible cane (ROC 22) to a rate above 20%. The tested canes are 3rd year ratoon canes. Liquid-cultured pJMT (pre-treated with IPTG; the concentration of pJMT fermentation product to the soil is approximately 4 × 10^5^ cell/cm^2^) was applied within three days from germination of ratoons after cutting off the above-ground part of the canes, following the established procedure [24].

## 3. Results

### 3.1. MeJA Suppresses S. scitamineum Mating/Filamentation

**Figure 1:**
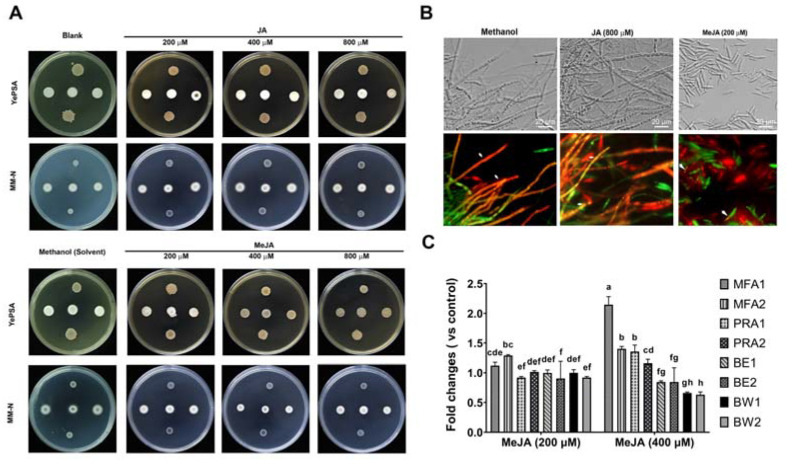
MeJA suppressed *S. scitamineum* dikaryotic hyphae growth after sexual mating. (**A**) The *MAT-1* (*eGFP*) and *MAT-2* (*dsRED*) sporidia were cultured in liquid YePS medium for 2 d and adjusted to OD_600_ ≈ 1.0, before mixed as 1:1 ratio and inoculated on YePSA or MM-N medium containing the indicated chemicals of various concentrations. The mating cultures were kept in the dark, at 28 °C, for 5 d before photographing. *MAT-1* sporidia were spotted in the upper panel, while *MAT-2* in the lower panel, of each plate. Three colonies in the middle of each plate are the mixed *MAT-1* and *MAT-2* sporidia to induce mating and filamentation. (**B**) Microscopic observation of fluorescent protein-tagged *S. scitamineum MAT-1* and *MAT-2* sporidia [19] during sexual mating/filamentous growth, using ZEISS Observer Z1. Arrows denote un-mating sporidia or hyphae. Scale bars = 20 μm. (**C**) qPCR analysis to assess transcription of a locus gene *MFA* 1/2 and *PRA* 1/2, and b locus *BE* 1/2 and *BW* 1/2 during *S. scitamineum* filamentation with or without MeJA treatment. The 1:1 ratio mixed *MAT-1* and *MAT-2* sporidia was allowed to grow on YePS medium under 28 °C, for 2 d, before total RNA extraction and qRT-PCR analysis. Relative gene expression level was calculated by the 2^−ΔΔCT^ method [22] with *ACTIN* as an internal control. Primers used for transcriptional profiling were listed in Table 1. Different letters indicate significant differences, *n* = 3. Methanol (20 μL per 40 mL medium) served as solvent control in (**A**–**C**). The experiments were repeated three times.

We first tested the effect of MeJA, a phytohormone used by plants against biotic or abiotic stresses [12], on *S. scitamineum* mating/filamentation, which is a critical step determining fungal pathogenicity. The result showed that, with 400–800 μM of MeJA, the filamentation (dikaryotic hyphae growth) of *S. scitamineum* was completely suppressed, and 200 μM MeJA caused reduced filamentation (Figure 1A). In contrast, treatment of JA, the precursor of MeJA, did not affect *S. scitamineum* filamentation even under high concentration (800 μM; Figure 1A). We conclude that MeJA specifically suppressed *S. scitamineum* dikaryotic hyphae growth.

We further assessed whether MeJA affected sexual mating by observing sporidia conjugation between two fluorescent proteins tagged sporidia of opposite mating types, and the following dikaryotic hyphae formation [19]. Under MeJA treatment, we observed abundant sporidia staying as monocyte status, failing to conjugate with the sporidia of the opposite mating type even though they were close to each other (Figure 1B). In contrast, the untreated or the JA-treated sporidia can form the healthy hyphal, displaying mixed fluorescent signals, suggesting cellular fusion between two sporidia (Figure 1B). This confirms that MeJA is able to suppress *S. scitamineum* sexual mating. We further measured the transcriptional levels of a and b locus genes, which control sexual mating and filamentous growth, respectively [25], under different treatment conditions. The results showed that the pheromone genes *mfa*1/2 and pheromones receptor gene *pra*1/2 increased their transcription significantly in the presence of MeJA. In contrast, the transcription levels of b locus genes controlling filamentous growth and pathogenicity were suppressed by MeJA (Figure 1C), likely accounting for reduced filamentous growth. Overall, MeJA is a phytohormone with potential in suppressing the pathogenic development of the sugarcane smut fungus.

### 3.2. MeJA Suppresses Maize Smut Disease Symptom in Pot Experiment

**Figure 2:**
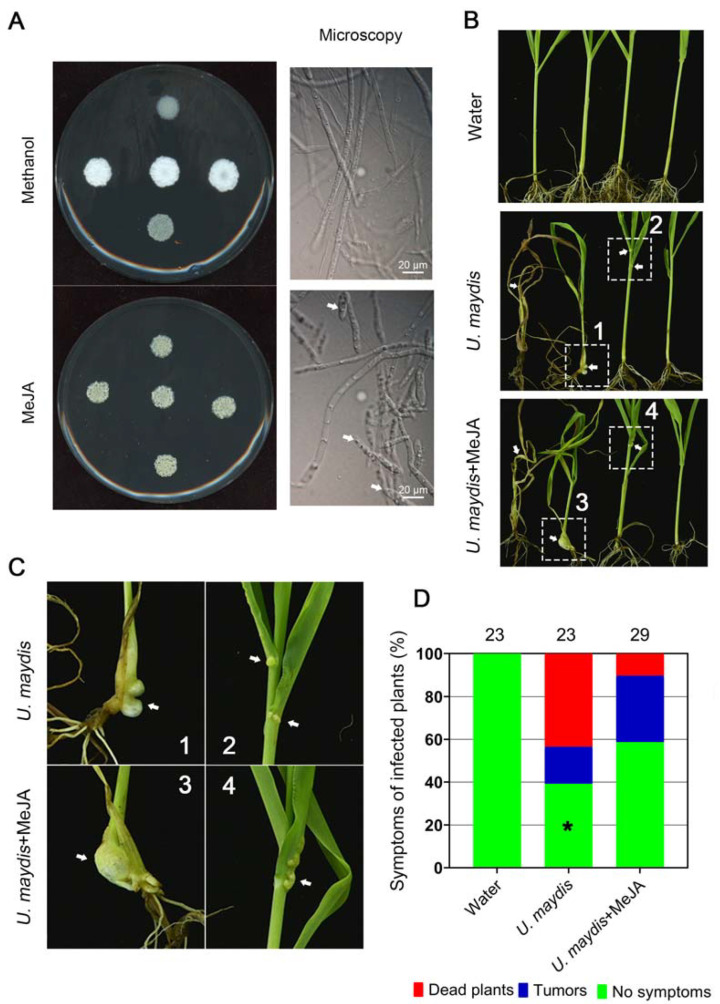
MeJA suppressed *U. maydis* sexual mating/filamentation. (**A**) The *MAT-1* and *MAT-2* sporidia of OD_600_ ≈ 1.0 were mixed as 1:1 (*v*/*v*) and inoculated on the YePSA medium containing MeJA (400 μM) or methanol (20 μL per 40 mL medium; solvent control). The mating cultures were kept in the dark, at 28 °C, for 5 d before photographing and microscopic observation. Scale bar = 20 μm. (**B**–**D**): Mixed *U. maydis* sporidia (1 mL; OD_600_ ≈ 1.0; *MAT-1: MAT-2*= 1:1, *v*/*v*), with or without MeJA (400 μM) treatment, were injected to maize seedlings at the four-leaf stage (Hua Meitian 9; DYQS-16 × TG-9). Injection with water served as blank control. The injected seedlings were allowed to grow in pots under natural conditions for 21 d, before photographing the tumor symptoms, as denoted by the arrows. The histogram was drawn by GraphPad Prism (Version: 8.0.2). The experiments were repeated three times, containing at least 23 seedlings for each treatment. Significant differences in no-symptom plants between *U. maydis*-infected seedlings with or without MeJA treatment were determined by a two-tailed Student’s *t*-test (* *p* = 0.0175).

As it takes a long time for the systematic infection of *S. scitamineum* on sugarcane before whip symptoms develop, we instead used maize smut fungus *U. maydis* to test the effect of MeJA on fungal pathogenicity. *U. maydis* is a model fungus belonging to basidiomycetes and closely related to *S. scitamineum*. As it takes long time for typical “whip” symptoms to develop when caused by *S. scitamineum* infection to the sugarcane, processing a timely application of MeJA would be difficult. Here, we intend to test the potential effect of MeJA on smut disease suppression by using the *U. maydis*-maize system. First, we tested the effect of MeJA on *U. maydis* sexual mating and filamentous growth. As is similarly observed in *S. scitamineum*, MeJA was effective in suppressing *U. maydis* filamentous growth after sexual mating (Figure 2A). Overall, MeJA caused reduced dimorphic switch in *U. maydis*.

We next tested the effect of MeJA on controlling maize smut disease. Approximately 60.87% of *U. maydis* infected seedlings displayed tumor symptoms on the stems or leaves, and some seedlings were even dead (Figure 2B,C). MeJA treatment could effectively reduce tumor formation rate, to 41.38% (Figure 2D). The death rate of *U. maydis*-infected seedling under MeJA treatment was significantly reduced as compared to un-treated seedlings (Figure 2D). Therefore, we conclude that MeJA has potential in preventing smut disease caused by fungi.

### 3.3. Construction of Escherichia coli Expressing Plant JMT Gene

**Figure 3:**
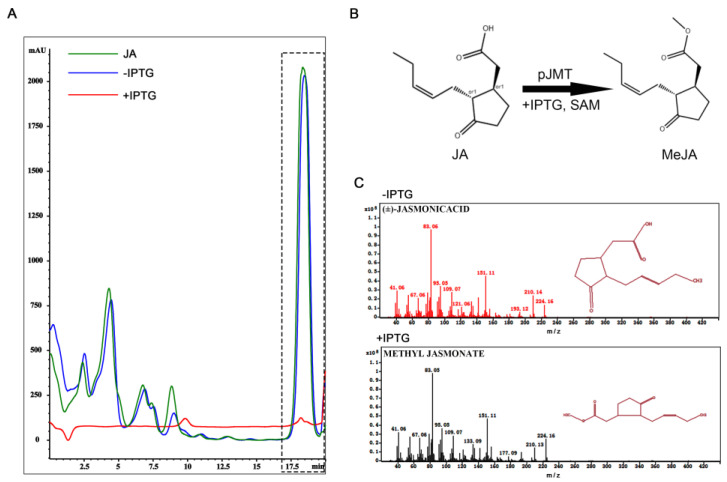
*E. coli* pJMT strain was able to convert JA to MeJA under in vitro culture conditions. (**A**) Detection of JA by HPLC. The region in the dashed box denotes JA, detected as retention time = 18.5 min, wavelength = 205 nm. The green line denotes JA standard, while the red line and green line, respectively, denote the supernatant extracted from the pJMT culture with or without IPTG induction. Peak area for the green line is 107,510.0, and 1815.1 for red line. (**B**) Schematic representation of pJMT catalyzing conversion of JA to MeJA under in vitro culture conditions. (**C**) GC-MS analysis to detect JA and MeJA from supernatant of the pJMT culture without IPTG induction (upper panel) or under IPTG induction (lower panel).

We next generated a plasmid for expressing the plant *JMT* gene (GenBank: AY008434.1), encoding the jasmonic acid carboxyl methyl transferase that catalyzes conversion from JA to MeJA [17]. The *JMT* gene was driven by a T7 promoter following the lacI repressor; therefore, it is inducible by isopropyl-β-D-thiogalactoside (IPTG) [21]. We transformed this constructed vector into *E. coli* (hereafter named as pJMT strain) and tested whether it could produce MeJA, by using JA as the precursor and SAM (S-adenosyl-L-methionine) as a methyl donor. Under in vitro culture conditions, IPTG induction led to a significant decrease in JA (Figure 3A, retention time ≈ 18.5 min) content detected in the supernatant of pJMT strain when compared to what was detected in the supernatant from un-treated pJMT strain (Figure 3A). This indicates that induced expression of *JMT* gene catalyzed conversion from JA to MeJA (Figure 3B). Furthermore, we used GC/MS to detect the presence of MeJA in the supernatant of pJMT strain, with or without IPTG inducion. The results showed that MeJA was detected in IPTG-induced pJMT strain, while not in un-induced strain (Figure 3C). Overall, the result showed that the induced JMT enzyme was capable of catalyzing conversion from JA to MeJA, likely using SAM as a methyl donor, under in vitro culture conditions.

**Figure 4:**
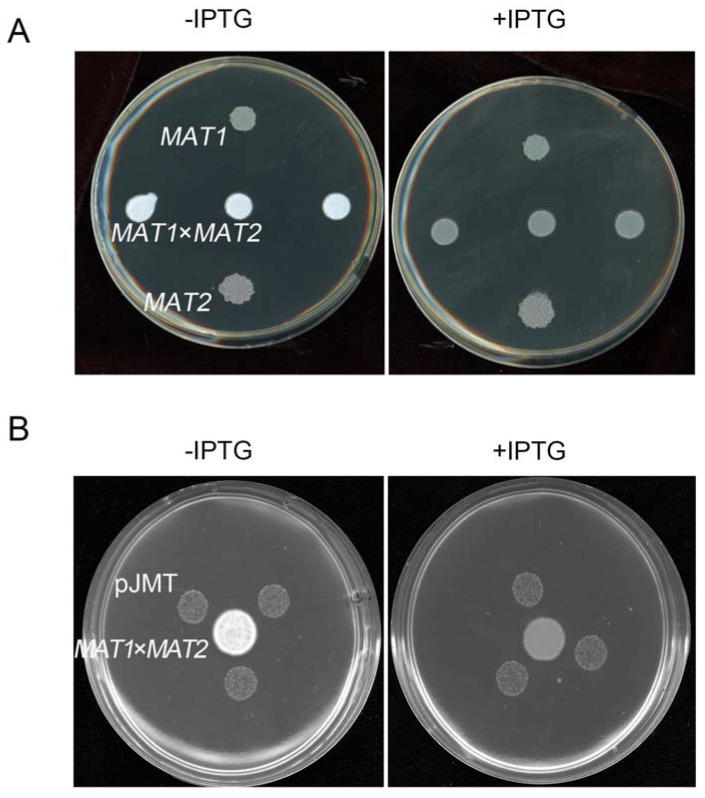
*E. coli* pJMT strain suppressed *S. scitamineum* filamentation under in vitro culture conditions. The pJMT strain was able to suppress filamentous growth of *S. scitamineum* after sexual mating by supplying the supernatent to the fungal colonies (**A**), or in confrontation assay (**B**). (**A**) JA was added in the liquid LB to reach a final concentration of 200 μM, with or without IPTG (1 mM) to induce *JMT* induction. A total of 4 mL supernatant of pJMT culture was mixed with 6 mL YePS medium, on which *MAT-1*, *MAT-2* sporidia, or mixed sporidia (to induce mating and filamentation) were allowed to grow for 3 d before photographing. (**B**) A total of 200 μM JA was added into YePS medium, on which mating *MAT-1* × *MAT-2* culture was confronted with three colonies of pJMT, with or without IPTG induction. The results were observed after 3 d of incubation.

We further tested whether the pJMT strain was able to suppress *S. scitamineum* filamentous growth by producing MeJA. By either confrontation, or supplying the supernatant of pJMT culture, the dikaryotic hyphae growth of *S. scitamineum* was significantly suppressed, when the *JMT* gene was induced by IPTG (Figure 4A,B). This indicates that the pJMT strain was able to produce and secrete MeJA, and has potential as a biocontrol agent against the sugarcane smut fungus.

### 3.4. Utilization of E. coli pJMT Strain in Controlling Smut Diseases

**Figure 5:**
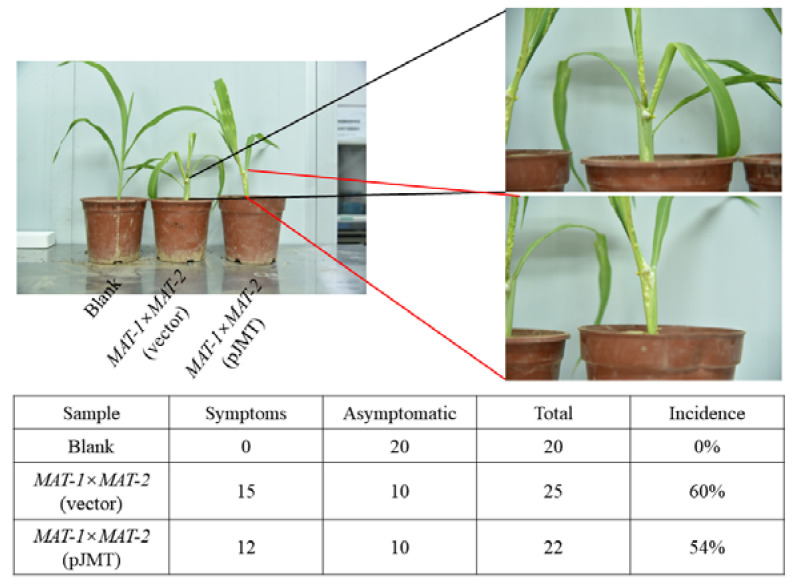
*E. coli* pJMT applied in controlling maize smut disease in pot experiment. The *U. maydis MAT-1* and *MAT-2* sporidia (1:1, *v/v*, OD_600_ ≈ 1.0) were mixed, and 1 mL of such mixture was injected into the the stem of four-leaf stage maize seedlings. In the treatment group, the *U. maydis* sporidia mixture was suspended with liquid-cultured pJMT (OD_600_ ≈ 1.0). The *E. coli* harboring the vector pRSFDUET-1 served as an untreated control, and the seedlings injected with water as the blank control. The tumor symptoms were examined and documented at 21 d post infection.

We next tested the effect of pJMT strain in controlling smut diseases, caused by *U. maydis* on maize or by *S. scitamineum* on sugarcane. As sugarcane smut caused by *S. scitamineum* takes a longer time (usually 3–6 months) to develop the typical “whip” symptom, we firstly tested the potential disease control capacity of pJMT on maize seedlings grown in pots. The results showed that *U. maydis* infection caused tumors formed on the stem of maize seedlings, either treated with pJMT strain or the control strain carrying vector only (Figure 5). The disease incidence rate seems to show no obvious difference between pJMT or the control strain-treated plants (Figure 5). Therefore, the pJMT strain did not seem to be effective in suppressing tumor formation on the maize stems or leaves.

**Figure 6:**
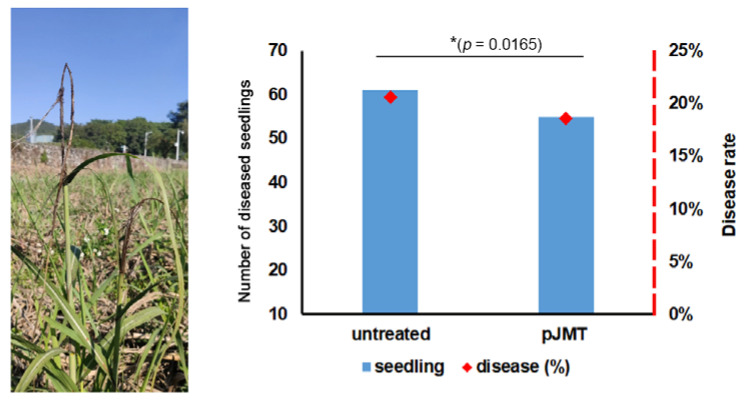
Black whip symptom of sugarcane smut in the sugarcane with or without pJMT treatment under field conditions. Ratoons of the canes grown in the diseased field in the experimental fields of South China Agricultural University (23.23 E, 113.63 W) were used for testing smut control capacity of pJMT strain. Three plots containing approximately 290 seedlings, out of 90 clumps, were, respectively, set as control or pJMT treatment plots. The pJMT culture was pre-treated with IPTG before being applied to the ratoons within 3 days after cutting the above-ground parts. Disease symptoms, including typical symptom and obvious growth defects of the infected seedlings, were evaluated at three months post growth of the ratoons. A seedling with typical black whip and slim stem symptoms is displayed in left panel. The disease occurring rate based on the number of black whips is quantified and presented in the right panel. A significant difference (* *p* = 0.0165) in the number of diseased seedlings in comparison between the pJMT treatment and untreated (control) plot was determined by a two-tailed Student’s *t*-test.

Under laboratory conditions, pJMT strain showed a significant inhibitory effect on the dimorphic switch of *S. scitamineum* (Figure 4B). We assumed that it could be a potential biocontrol agent for protecting sugarcane from smut disease. A field experiment was conducted to evaluate the control ability of pJMT strain against sugarcane smut. Approximately 290 sugarcanes from 90 clumps were grown from ratoons in an established diseased field and used for untreated control and pJMT treatment plots, respectively. Liquid-cultured pJMT (pre-treated with IPTG; the concentration of pJMT fermentation product to the soil is approximately 4 × 10^5^ cell/cm^2^) were applied within three days from the germination of ratoons. The disease canes grew short and slim, and the whips appeared from the trunks of a very early stage, in either untreated or pJMT-treated plots (Figure 6). There was no significant difference in the disease-occurring rate between the untreated control plot (20.6% diseased seedlings out of a total 294 seedlings) and the pJMT strain treatment (18.6% diseased out of a total of 294 seedlings).

In summary, the engineered *E. coli* pJMT strain—which can catalyze a JA-to-MeJA conversion—displayed a mild suppression effect on maize smut in the pot experiment and had no obvious effect on sugarcane smut under field conditions. This strain needs further optimization before it can be applied as a biocontrol agent against smut diseases.

## 4. Discussion

Sugarcane smut caused by *Sporisorium scitamineum* is considered as the most serious and widespread disease of sugarcane [2], severely affecting sugarcane yields and quality, and thus causes significant economic loss [26,27,28]. The disease control methods for sugarcane smut include physical control, fungicide control, and disease-resistant breeding [29,30,31]. The application of fungicides is not only expensive, but also leads to serious environmental pollution problems and drug-resistance to the pathogens with increasing fungicide doses in pursuit of the desired control effects. Although disease-resistant breeding is the most effective method to control sugarcane smut in agricultural practices, the breeding process of disease-resistant varieties is difficult and time-consuming [32,33], and ever-evolving pathogens cause the loss of resistance of disease-resistant varieties [34]. At present, a highly efficient, sustainable, and environmentally friendly control strategy for sugarcane smut is still lacking, and is in urgent need.

Phytohormones not only regulate plant growth, but also precipitate/regulate plant immune responses to pathogens. Auxin regulates plant cell division, enlargement, and differentiation [35]. Exogenous addition of auxin increases the tolerance of *Medicago truncatula* to *Macrophomina phaseolina* infection [36], indicating a positive regulation of plant immunity. In *Arabidopsis thaliana*, the exogenous addition of auxin affects the proliferation of *Pseudomonous syringae* [37], but does not affect *Fusarium oxysporum* [38]. Salicylic acid (SA) and jasmonic acid (JA) are important defense hormones. Necrotrophic pathogens can activate JA-related defense responses [39]. SA plays an important role in defending against infection by biotrophic and hemibiotroph pathogens [40]. Moreover, as a plant hormone, SA can interact with a variety of plant hormone-related signaling pathways to activate the immune response and disease resistance of plants [41]. The application of MeJA to roots or foliar tissues can enhance transcriptional expression levels of resistance genes in sugarcane towards various classes of soil-borne pests and pathogens [42]. MeJA increases plant resistance by regulating antioxidant defense systems in sugarcane seedlings [43], *Panax ginseng* C.A. Mey. roots [44], and *Glycine max* L. leaves [45]. Furthermore, foliar application of MeJA could improve the grape and wine quality [46].

In this study, we found that the phytohormone MeJA could inhibit the dimorphic transition, a critical step in pathogenic development, of smut fungi *S. scitamineum* and *U. maydis* (Figure 1 and Figure 2A). The a and b locus play an important role in sexual mating and hypha formation [3,5,24]. The qRT-PCR results of the coding genes showed that MeJA inhibits *S. scitamineum* sexual mating while causing a significant increase in the transcriptional levels of the mating type genes, pheromone genes mfa1/2, and pheromones receptor gene pra1/2. We infer that suppressed transcription of b locus genes is more important for regulating filamentation, which is inhibited by MeJA. Up-regulation of the a locus genes does not guarantee promoted sexual mating, as the encoded pheromone peptides need post-translational modification [8] for activation, and the pheromone receptors Pra1/2 need to recognize the activated pheromone peptide from the opposite mating-type [47]. In addition, we noticed a limited difference in the transcription of the a and b locus genes in presence of MeJA, which would almost completely suppress mating/filamentous growth. We hypothesize that in addition to suppression of a and b locus gene transcription, MeJA may target other pathways critical to the filamentation of *S. scitamineum*, which were not identified in this study.

Furthermore, we found that the exogenous addition of MeJA suppressed disease symptoms (tumors formation) caused by a *U. maydis* infection in maize seedlings, under the pot experiment (Figure 2B). The pJMT strain, heterogeneously expressing the plant *JMT* gene, was able to catalyze MeJA production using JA as a precursor (Figure 3). Although, under in vitro culture conditions, the formation of *S. scitamineum* dikaryotic hyphae could be effectively inhibited by confrontation or by supplying the supernatant of the pJMT culture (Figure 4), suggesting that such a MeJA-producing pJMT strain could be potentially used as a biocontrol agent against smut diseases caused by the fungal pathogens. However, direct application of this pJMT strain to the infected maize or sugarcane seedlings, in pot experiments or in field experiments, did not show an obvious disease control ability (Figure 5 and Figure 6). When performing the pot and field experiments, we pre-treated the pJMT culture with IPTG to induce JMT expression. A possible reason for the failure of the pJMT strain in controlling smut diseases may be the fact that the symptom development process in the field is long and complicated. In the future, we would like to further modify this strain by using a strong, constitutive, and native promoter of *E. coli* to drive the JMT gene. Alternatively, a pathogen-inducible promoter would be screened and tested.

In conclusion, our results demonstrate that the phytohormone MeJA could suppress a dimorphic switch, which was established as a key step of pathogenicity for smut fungi (*S. scitamineum* and *U. maydis*). We further constructed a pJMT strain by expressing the plant’s *JMT* gene, and verified that the engineered strain could catalyze a conversion from JA to MeJA. At present, this pJMT construct showed no capacity in suppressing smut disease either in maize (pot condition) or sugarcane (field condition). However, the idea that a bacteria strain expressing a JMT gene could utilize a plant-source JA to facilitate MeJA production was shown to suppress the dikaryotic hyphae growth of smut fungi in this study. Future attempts will be conducted to monitor and enhance the colonization ability and/or strain stability of the pJMT strain, with the aim to develop it as a useful tool in the prevention and management of smut diseases.

## Figures and Tables

**Table 1:** Primer sequences.

Primer	Sequence (5′-3′)	Notes
Actin-F	CAGCTCGATGAAGGTCAAGAT	qRT-PCR
Actin-R	CAGCTCGATGAAGGTCAAGAT	
Q-bE1-F	CCAACGACGAAAGCGCGACG	
Q-bE1-R	GACTCTCTGCGAGCGGGCAT	
Q-bE2-F	CCAACGACGAAAGCGCGACG	
Q-bE2-R	GACTCTCTGCGAGCGGGCAT	
Q-bW1-F	CGAGAAAGGCACACAACGTC	
Q-bW1-R	CACCTTTTGGGGAGTTCCGA	
Q-bW2-F	TGTTGATGAGCCAGTGCCTT	
Q-bW2-R	AGTTCCGACTGGCTGAAGTG	
Pra2-P1F	GAAGAGCCTCAGCCGTTATAC	
Pra2-P1R	GGGTTCCCTTACTGAACCTTAG	
Q-PCR-Mfa1-F	ATGCTTTCCATCTTTACCCAGA	
Q-PCR-Mfa1-R	GTGCAGCTAGAGTAGCCAAG	
Mfa2-P1F	CGTCCAGGCCATTGTTTCT	
Mfa2-P1R	TAGGCCACGGTGCAGTA	
Q-PCR-Pra1-F	GGACGCTATCACCCAATCTTAC	
Q-PCR-Pra1-R	TCTCCAACATGGCAACACTC	
pJMT-F	GAATTCATGGAGGTAATGCGA	Red front denotes restriction enzyme sites used for plasmid construction
pJMT-R	GTCGACTCAACCGGTTCTAAC	

## Data Availability

No data available.

## References

[1] Bhuiyan S.A., Magarey R.C., McNeil M.D., Aitken K.S. (2021). Sugarcane Smut, Caused by *Sporisorium scitamineum*, a Major Disease of Sugarcane: A Contemporary Review. Phytopathology.

[2] Rajput M.A., Rajput N.A., Syed R.N., Lodhi A.M., Que Y. (2021). Sugarcane Smut: Current Knowledge and the Way Forward for Management. J. Fungi.

[3] Agisha V.N., Nalayeni K., Ashwin N.M.R., Vinodhini R.T., Jeyalekshmi K., Suraj Kumar M., Ramesh Sundar A., Malathi P., Viswanathan R. (2022). Molecular Discrimination of Opposite Mating Type Haploids of *Sporisorium scitamineum* and Establishing Their Dimorphic Transitions During Interaction with Sugarcane. Sugar Tech..

[4] Zuo W., Okmen B., Depotter J.R.L., Ebert M.K., Redkar A., Misas Villamil J., Doehlemann G. (2019). Molecular Interactions Between Smut Fungi and Their Host Plants. Annu. Rev. Phytopathol..

[5] Kijpornyongpan T., Aime M.C. (2020). Investigating the Smuts: Common Cues, Signaling Pathways, and the Role of MAT in Dimorphic Switching and Pathogenesis. J. Fungi.

[6] Spellig T., Bolker M., Lottspeich F., Frank R.W., Kahmann R. (1994). Pheromones trigger filamentous growth in *Ustilago maydis*. Embo J..

[7] Yan M., Dai W., Cai E., Deng Y.Z., Chang C., Jiang Z., Zhang L.H. (2016). Transcriptome analysis of *Sporisorium scitamineum* reveals critical environmental signals for fungal sexual mating and filamentous growth. BMC Genom..

[8] Sun S., Deng Y., Cai E., Yan M., Li L., Chen B., Chang C., Jiang Z. (2019). The Farnesyltransferase Beta-Subunit Ram1 Regulates *Sporisorium scitamineum* Mating, Pathogenicity and Cell Wall Integrity. Front. Microbiol..

[9] Deng Y.Z., Zhang B., Chang C.Q., Wang Y.X., Lu S., Sung S.Q., Zhang X.M., Chen B.S., Jiang Z.D. (2018). The MAP Kinase SsKpp2 Is Required for Mating/Filamentation in *Sporisorium scitamineum*. Front. Microbiol..

[10] Chang C., Cai E., Deng Y.Z., Mei D., Qiu S., Chen B., Zhang L.H., Jiang Z. (2019). cAMP/PKA signalling pathway regulates redox homeostasis essential for *Sporisorium scitamineum* mating/filamentation and virulence. Environ. Microbiol..

[11] Cai E., Li L., Deng Y., Sun S., Jia H., Wu R., Zhang L., Jiang Z., Chang C. (2021). MAP kinase Hog1 mediates a cytochrome P450 oxidoreductase to promote the *Sporisorium scitamineum* cell survival under oxidative stress. Environ. Microbiol..

[12] Mousavi S.R., Niknejad Y., Fallah H., Tari D.B. (2020). Methyl jasmonate alleviates arsenic toxicity in rice. Plant Cell Rep..

[13] Krol P., Igielski R., Pollmann S., Kepczynska E. (2015). Priming of seeds with methyl jasmonate induced resistance to hemi-biotroph *Fusarium oxysporum* f.sp. *lycopersici* in tomato via 12-oxo-phytodienoic acid, salicylic acid, and flavonol accumulation. J. Plant Physiol..

[14] Farooq M.A., Gill R.A., Islam F., Ali B., Liu H., Xu J., He S., Zhou W. (2016). Methyl Jasmonate Regulates Antioxidant Defense and Suppresses Arsenic Uptake in *Brassica napus* L.. Front. Plant Sci..

[15] Thomma B.P.H.J., Eggermonta K., Broekaerta W.F., Cammueab B.P.A. (2000). Disease development of several fungi on *Arabidopsis* can be reduced by treatment with methyl jasmonate. Plant Physiol. Biochem..

[16] Santino A., Taurino M., De Domenico S., Bonsegna S., Poltronieri P., Pastor V., Flors V. (2013). Jasmonate signaling in plant development and defense response to multiple (a)biotic stresses. Plant Cell Rep..

[17] Seo H.S., Song J.T., Cheong J.J., Lee Y.H., Lee Y.W., Hwang I., Lee J.S., Choi Y.D. (2001). Jasmonic acid carboxyl methyltransferase: A key enzyme for jasmonate-regulated plant responses. Proc. Natl. Acad. Sci. USA..

[18] Qi J., Li J., Han X., Li R., Wu J., Yu H., Hu L., Xiao Y., Lu J., Lou Y. (2016). Jasmonic acid carboxyl methyltransferase regulates development and herbivory-induced defense response in rice. J. Integr. Plant Biol..

[19] Yan M., Cai E., Zhou J., Chang C., Xi P., Shen W., Li L., Jiang Z., Deng Y.Z., Zhang L.H. (2016). A Dual-Color Imaging System for Sugarcane Smut Fungus *Sporisorium scitamineum*. Plant Dis..

[20] Yang B., Zheng P., Wu D., Chen P. (2021). Efficient Biosynthesis of Raspberry Ketone by Engineered *Escherichia coli* Coexpressing Zingerone Synthase and Glucose Dehydrogenase. J. Agric. Food Chem..

[21] Zhang C., Liu L., Teng L., Chen J., Liu J., Li J., Du G., Chen J. (2012). Metabolic engineering of *Escherichia coli* BL21 for biosynthesis of heparosan, a bioengineered heparin precursor. Metab. Eng..

[22] Livak K.J., Schmittgen T.D. (2001). Analysis of relative gene expression data using real-time quantitative PCR and the 2(-Delta Delta C(T)) Method. Methods..

[23] Huang N., Ling H., Su Y., Liu F., Xu L., Su W., Wu Q., Guo J., Gao S., Que Y. (2018). Transcriptional analysis identifies major pathways as response components to *Sporisorium scitamineum* stress in sugarcane. Gene..

[24] Cui G., Yin K., Lin N., Liang M., Huang C., Chang C., Xi P., Deng Y.Z. (2020). *Burkholderia gladioli* CGB10: A Novel Strain Biocontrolling the Sugarcane Smut Disease. Microorganisms..

[25] Yan M., Zhu G., Lin S., Xian X., Chang C., Xi P., Shen W., Huang W., Cai E., Jiang Z. (2016). The mating-type locus b of the sugarcane smut *Sporisorium scitamineum* is essential for mating, filamentous growth and pathogenicity. Fungal. Genet. Biol..

[26] Comstock J.C. (1987). Sugarcane Smut: Comparison of Natural Infection Testing and Artificial Inoculation. Hawaii. Plant. Rec..

[27] Khan H.M.W.A., Chattha A.A., Munir M., Zia A. (2009). Evaluation of resistance in sugarcane promising lines against whip smut. Pak. J. Phytopathol..

[28] Tadesse A. (2009). Increasing Crop Prodcution through Improved Plant Protection.

[29] Ming R., Moore P.H., Wu K.K., D’Hont A., Glaszmann J.C., Tew T.L., Mirkov T.E., da Silva J., Jifon J., Rai M. (2010). Sugarcane Improvement through Breeding and Biotechnology. Plant Breed. Rev..

[30] Sundar A.R., Barnabas E.L., Malathi P., Viswanathan R. (2012). A Mini-Review on Smut Disease of Sugarcane Caused by *Sporisorium scitamineum*. InTech Open..

[31] Shailbala, Sharma S.K. (2013). Effect of fungicides and hot water treatment on control of sugarcane smut. Pestology.

[32] Silva J.A., Sorrells M.E., Burnquist W.L., Tanksley S.D. (1993). RFLP linkage map and genome analysis of *Saccharum spontaneum*. Genome.

[33] Burnquist W.L., Sorrelles M.E. (1995). Characterization of Genetic Variability in Saccharum germplasm by Means of Restriction Fragment Length Polymorphism (RFLP) Analysis.

[34] Nelson R., Wiesner-Hanks T., Wisser R., Balint-Kurti P. (2018). Navigating complexity to breed disease-resistant crops. Nat. Rev. Genet..

[35] Sundberg E., Ostergaard L. (2009). Distinct and dynamic auxin activities during reproductive development. Cold Spring Harb. Perspect. Biol..

[36] Mah K.M., Uppalapati S.R., Tang Y.H., Allen S., Shuai B. (2012). Gene expression profiling of *Macrophomina phaseolina* infected *Medicago truncatula* roots reveals a role for auxin in plant tolerance against the charcoal rot pathogen. Physiol. Mol. Plant Pathol..

[37] Chen Z., Agnew J.L., Cohen J.D., He P., Shan L., Sheen J., Kunkel B.N. (2007). *Pseudomonas syringae* type III effector AvrRpt2 alters *Arabidopsis thaliana* auxin physiology. Proc. Natl. Acad. Sci. USA..

[38] Kidd B.N., Kadoo N.Y., Dombrecht B., Tekeoglu M., Gardiner D.M., Thatcher L.F., Aitken E.A., Schenk P.M., Manners J.M., Kazan K. (2011). Auxin signaling and transport promote susceptibility to the root-infecting fungal pathogen *Fusarium oxysporum* in *Arabidopsis*. Mol. Plant Microbe Interact..

[39] Katagiri F., Tsuda K. (2010). Understanding the plant immune system. Mol. Plant Microbe Interact..

[40] Al-Daoude A., Al-Shehadah E., Shoaib A., Jawhar M., Arabi M.I.E. (2019). Salicylic Acid Pathway Changes in Barley Plants Challenged with either a Biotrophic or a Necrotrophic Pathogen. Cereal. Res. Commun..

[41] An C., Mou Z. (2011). Salicylic acid and its function in plant immunity. J. Integr. Plant Biol..

[42] Bower N.I., Casu R.E., Maclean D.J., Reverter A., Chapman S.C., Manners J.M. (2005). Transcriptional response of sugarcane roots to methyl jasmonate. Plant Sci..

[43] Seema G., Srivastava M.K., Srivastava S., Shrivastava A.K. (2003). Effect of methyl jasmonate on sugarcane seedlings. Sugar Tech..

[44] Ali M.B., Yu K.W., Hahn E.J., Paek K.Y. (2006). Methyl jasmonate and salicylic acid elicitation induces ginsenosides accumulation, enzymatic and non-enzymatic antioxidant in suspension culture *Panax ginseng* roots in bioreactors. Plant Cell Rep..

[45] Keramat B., Kalantari K., Arvin M. (2009). Effects of methyl jasmonate in regulating cadmium induced oxidative stress in soybean plant (*Glycine max* L.). Afr. J. Microbiol. Res..

[46] Garde-Cerdan T., Portu J., Lopez R., Santamaria P. (2016). Effect of methyl jasmonate application to grapevine leaves on grape amino acid content. Food Chem..

[47] Lu S., Shen X., Chen B. (2017). Development of an efficient vector system for gene knock-out and near in-*cis* gene complementation in the sugarcane smut fungus. Sci. Rep..

